# A Metabolomic Strategy to Screen the Prototype Components and Metabolites of *Shuang-Huang-Lian Injection* in Human Serum by Ultra Performance Liquid Chromatography Coupled with Quadrupole Time-of-Flight Mass Spectrometry

**DOI:** 10.1155/2014/241505

**Published:** 2014-02-26

**Authors:** Mingxing Guo, Baosheng Zhao, Haiyu Liu, Li Zhang, Long Peng, Lingling Qin, Zhixin Zhang, Jian Li, Chengke Cai, Xiaoyan Gao

**Affiliations:** ^1^Science Experiment Center for Traditional Chinese Medicine, Beijing University of Chinese Medicine, No. 11, North Third Ring Road, Chaoyang District, Beijing 100029, China; ^2^School of Basic Medical Sciences, Beijing University of Chinese Medicine, No. 11, North Third Ring Road, Chaoyang District, Beijing 100029, China; ^3^School of Chinese Material Medica, Beijing University of Chinese Medicine, South of Wangjing Middle Ring Road, Chaoyang District, Beijing, 100102, China

## Abstract

*Shuang-huang-lian injection* (*SHLI*) is a famous Chinese patent medicine, which has been wildly used in clinic to treat acute respiratory tract infection, pneumonia, influenza, and so forth. Despite the widespread clinical application, the prototype components and metabolites of *SHLI* have not been fully elucidated, especially in human body. To discover and screen the constituents or metabolites of Chinese medicine in biofluids tends to be more and more difficult due to the complexity of chemical compositions, metabolic reactions and matrix effects. In this work, a metabolomic strategy to comprehensively elucidate the prototype components and metabolites of *SHLI* in human serum conducted by UPLC-Q-TOF/MS was developed. Orthogonal partial least squared discriminant analysis (OPLS-DA) was applied to distinguish the exogenous, namely, drug-induced constituents, from endogenous in human serum. In the S-plot, 35 drug-induced constituents were found, including 23 prototype compounds and 12 metabolites which indicated that *SHLI* in human body mainly caused phase II metabolite reactions. It was concluded that the metabolomic strategy for identification of herbal constituents and metabolites in biological samples was successfully developed. This identification and structural elucidation of the chemical compounds provided essential data for further pharmacological and pharmacokinetics study of *SHLI*.

## 1. Introduction

*Shuang-huang-lian injection (SHLI)* is a typical Chinese herbal injection that is made from the extracts of *Flos Lonicerae Japonicae, Radix Scutellariae,* and *Fructus Forsythiae*. It has been widely used for the treatment of acute upper respiratory tract infections [1, 2]. Baicalin, chlorogenic acid, and forsythin are the marker compounds representing *Radix Scutellariae*, *Flos Lonicerae Japonicae,*   and *Fructus Forsythiae*, respectively, for the quality control of this medicine [3]. Though several published papers have reported the determination of major active components and metabolites in *Shuang-huang-lian (SHL) *preparations [4–6], there is no substantial evidence to confirm the holistic existing form of *SHLI* in vivo, especially in human body. Therefore, systematically, screening the constituents and metabolites of *SHLI *in human blood is of great significance for interpreting its material basis for pharmacological effects. Currently, the ingredients of *SHL* formula have been detected in rat blood [7]. However, the recent study suggests that species differences in key hepatic efflux transporters are sufficiently profound to warrant careful re-examination of conclusions and to design future studies with caution [8]. Some data have revealed that rat liver contains much more (~10-fold) apical multidrug resistance-associated protein 2 (Mrp2) resulting in a much higher capacity for the biliary excretion of organic anions in rats than human or other preclinical species [9]. Therefore, to reveal the pharmacological mechanism of *SHLI*, comprehensive analysis of the constituents and metabolites in human body is more scientific and rational.

The process of metabolite detection and identification is typically a labor-intensive and time-consuming process. This process has been simplified by the use of radiolabeled compounds and/or spectroscopic techniques such as mass spectrometry and NMR spectroscopy [10–13]. Of these analysis techniques, liquid chromatography coupled with electrospray ionization mass spectrometer has been widely used to detect and identify trace levels of drugs and metabolites in various biological samples due to its high sensitivity and selectivity [14–16]. Ultra performance liquid chromatography (UPLC) applied for short run times combined with a quadrupole/time of flight-mass spectrometer (Q/TOF-MS) which offers high mass accuracy has become a major tool that provides a significant source of global constituent and metabolite profiling data [17–19]. Given the chemical complexity of *SHLI* in vivo, UPLC-Q-TOF/MS provides faster separations for complex blood samples and valuable structural insights into the characterization of *SHLI* metabolites.

A straightforward approach for identifying exogenous metabolites in vivo is to compare the LC-MS chromatograms of biological samples collected before and after xenobiotic treatment. However, without using effective analysis method, it is difficult to identify exogenous metabolites through visual examination of LC-MS chromatograms that contain information from thousands of chemical species [20]. A metabolomic strategy has been developed to handle the acquired data and to search for the discriminating features from biosample sets. A xenobiotic and its metabolites only appear in the samples after xenobiotic treatment, and so when using metabolomic strategy, the differences between the control group and the xenobiotic-treated group are mainly defined by the presence of the xenobiotic and its metabolites. With appropriate data processing, the separation of the control group and the xenobiotic-treated group can be achieved in the score plot of a multivariate model, and exogenous metabolites can be conveniently identified by analyzing ions contributing to the separation of the two groups. Employing this approach, the present study aims to develop a metabolomic strategy to comprehensively elucidate the prototype components and metabolites of *SHLI* in human serum conducted by UPLC-Q-TOF/MS.

## 2. Experiment

### 2.1. Materials

*SHLI* was achieved from the Second Chinese Medicine Factory of Harbin Pharm. Group CO., Ltd. (No. 1204014). HPLC grade formic acid was obtained from Sigma Chemical Co., Ltd. (St. Louis, MO, USA). Methanol (HPLC grade) was acquired from Fisher Corporation (Michigan, USA). Water was purified with a Milli-Q system (Millipore, Bedford, USA).

### 2.2. Subjects and Clinical Trial Design

The study was approved by an independent ethics committee at Beijing University of Chinese Medicine, before recruitment commenced. Before the initiation of study procedures, all volunteers gave their written informed consent for participation in the study. Thirteen healthy volunteers, without taking any medication, participated in the study. They were aged between 25 and 40 years and with weight between 50 and 80 kg. After overnight fasting, early-morning blood samples (20 mL each) were collected from the medial cubital vein into evacuated tubes and marked as the control group (C group). Then participants were intravenous infusion of 60 mg/kg of *SHLI* (dissolved with 500 mL saline solution). The blood samples were collected at 0.5 h after *SHLI* administration and marked as *SHLI* dosed group (*SHLI* group). The blood supernatant was allowed to clot overnight at room temperature, and the clotted material was removed by centrifugation (3000 rpm, 15 min). The serum was collected and stored at −80°C.

### 2.3. Pretreatment Procedure for *SHLI*

The* Shuang-huang-lian* lyophilized powder for injection (0.1 g) was weighed and dissolved with 100 mL water. Then, it was filtered by a 0.22 *μ*m filter before UPLC-Q-TOF/MS analysis.

### 2.4. Pretreatment Procedure for Serum Samples

All serum samples were thawed at room temperature followed by methanol protein precipitation. Serum (200 *μ*L) was added with 600 *μ*L methanol, vortexed for 30 s, and centrifuged at 14000 g for 10 min at 4°C. Then, supernatant (400 *μ*L) was transferred to a clean tube and evaporated to dryness under a gentle stream of nitrogen. The residue was redissolved with 100 *μ*L ultra high purity water and transferred to an autosampler vial.

### 2.5. UPLC-Q-TOF/MS Analysis

Separation and detection of the components and metabolites of *SHLI* were performed on a Waters Acquit UPLC chromatographic system (Waters Corp., Milford, USA) equipped with a Evoe G2 Q/TOF (Waters MS Technologies, Manchester, UK). An electrospray ionization source (ESI) interface was used in both positive and negative ion modes. Acquit UPLC HSS T3 column (2.1 mm × 100 mm, 1.8 *μ*m, Waters, UK) was applied for all analyses. The mobile phase was composed of A (0.1% formic acid in water) and B (methanol) with a linear gradient elution: 0–1 min, maintained at 0% B; 1–5 min, from 0% B to 40% B; 5–8 min, from 40% B to 100% B; 8–13 min, maintained at 0% B; 13.0–13.1 min, isocratic of 0% B; 13.1–15 min, maintained at 0% B. The flow rate was 0.30 L/min. The analytic column and autosampler were maintained at temperatures of 45°C and 4°C, respectively. Then, 1 *μ*L of sample solution was injected for each run. Data were collected from *m/z* 50 to *m/z* 1200. For positive ion mode, the capillary and cone voltage were set at 3 kV and 35 V. For negative ion mode, the capillary and cone voltage were set at 2.5 kV and 35 V. The conservation gas was set at 700 L/h at a temperature of 350°C. The source temperature was set at 100°C. The cone gas was set at 50 L/h. Leucine-enkephalin was used as the lock mass solution to ensure the accuracy and reproducibility.

### 2.6. Data Processing and Statistical Analysis

The ES+ and ES− raw data was analyzed by MarkerLynx XS software (Waters Corp., Milford, USA). For extracting data from the raw file and detecting potential markers, the retention time range was set at 0–13 min, the mass range at 50–1000 amu, and the mass tolerance as 0.01. For detecting chromatographic peaks in the Apex Track Peak, peak width at 5% height was set at 1.00, and the peak-to-peak baseline noise was 0.00. For collecting parameters, the marker intensity threshold was set at 1000 cps, the mass window was 0.02 amu, and retention time window was 0.20 min. The noise elimination level was 6. This process provided alignment of drift (retention time and accurate mass) in data and ensured that a chromatographic peak was identified with the same parameters in each sample. Subsequently, a list of intensities or peak areas of the peaks was then generated for the first chromatogram, using the ER-*m/z* pairs as identifiers. The procedure was applied for each UPLC/MS analysis. The ion intensities or peak area for each peak detected was also normalized within each sample to the sum of the peak intensities in that sample. The three-dimensional data were introduced into the EZinfo 2.0 software (Waters Corporation, Milford, MA, USA) for orthogonal partial least-squares-discriminate analysis (OPLS-DA).

## 3. Results and Discussion

### 3.1. Identification and Analysis of Chemical Components in *SHLI*

**Figure 1:**
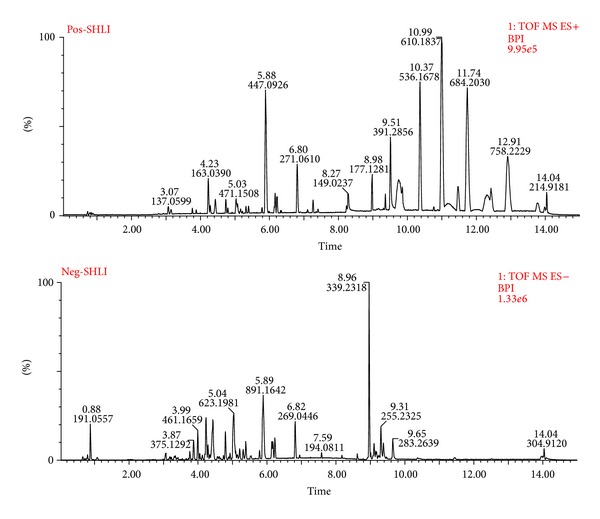
UPLC-Q-TOF/MS BPI chromatograms of *SHLI* in positive ion mode and negative ion mode.

Global profiling of both positive and negative ion modes was analyzed by UPLC-Q-TOF/MS. The typical base peak intensity (BPI) chromatograms (positive ion mode and negative ion mode) of *SHLI* were shown in Figure 1. In total, 38 constituents were detected and tentatively characterized in *SHLI* (Table 1). MS^E^ technique, a new technique used in deducing the splitting disciplinary of MS, was applied to data collection. MS^E^ technique could provide parallel alternating scans for acquisition at low collision energy to obtain precursor ion information or at a ramping of high collision energy to obtain a full-scan accurate mass of fragments, precursor ions, and neutral loss information [21, 22]. Here, the high precision MS/MS fragments information obtained from the MS^E^ technique were also listed in Table 1 to explain the structure information of the chemical constituents. All the constituents and the fragmentation information were consistent with previous reports [23, 24].

### 3.2. Analysis of Human Serum by Metabolomic Strategy

**Figure 2:**
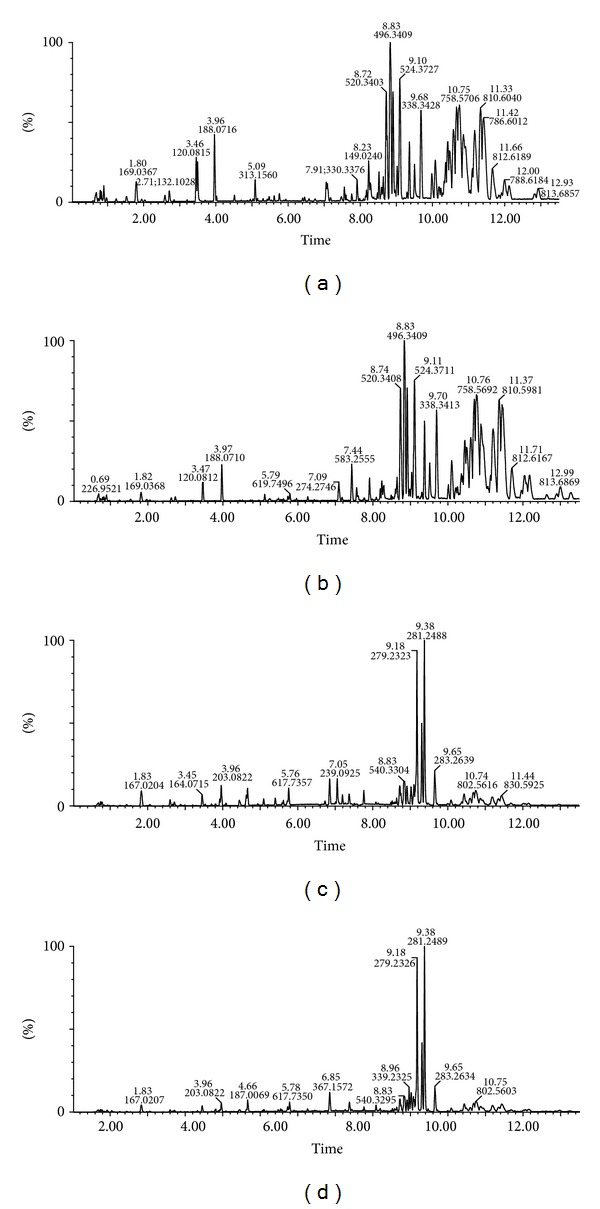
UPLC-Q-TOF/MS BPI chromatograms of human serum samples (a) before *SHLI* administration in positive ion mode, (b) after *SHLI* administration in positive ion mode, (c) before *SHLI* administration in negative ion mode, and (d) after *SHLI* administration in negative ion mode.

**Figure 3:**
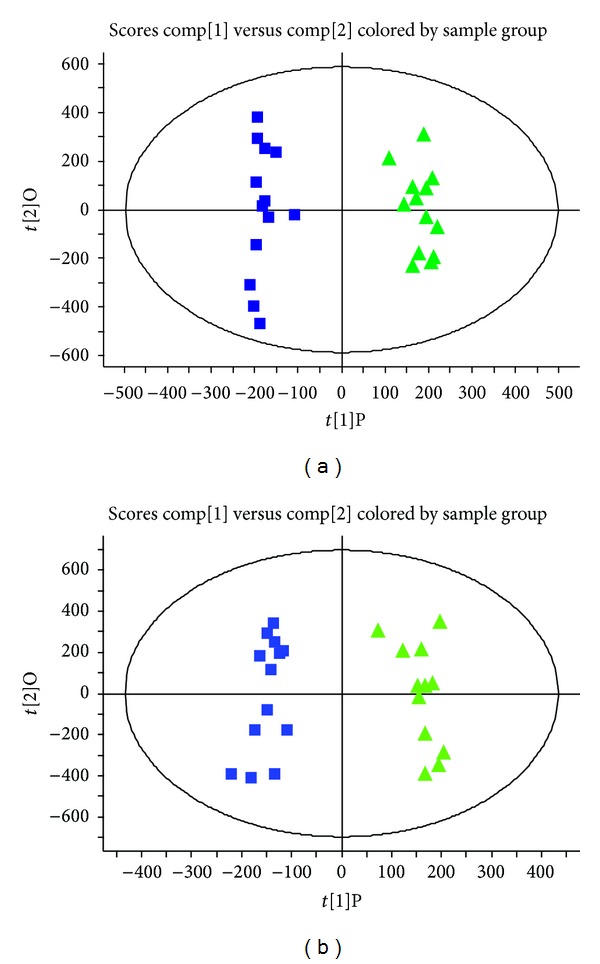
Score plots of OPLS-DA in human serum samples between C group (■) and *SHLI *group (▲) in (a) positive ion mode and (b) negative ion mode.

Figure 2 represented the typical BPI chromatograms (positive ion mode and negative ion mode) of human serum samples before and after *SHLI* administration. The prototype components and metabolites of *SHLI *in human serum were almost submerged by the endogenous metabolites due to the high level of endogenous signals. Interferences from biological matrices remain a major challenge to detection of metabolites in vivo. Without the presence of a radiolabeled isotope or a data-mining tool, it would be almost impossible to identify low level exogenous metabolites. In our work, a metabolomic strategy was employed to phenotype the differences between C group and *SHLI* group. The LC/MS data were processed using MarkerLynx XS to detect peaks and generate a three-dimensional data with *t*_*R*_-*m/z* pairs and the corresponding intensities. Statistical analysis by OPLS-DA was subsequently performed on the entire dataset.  Figure 3 showed the OPLS-DA score plots of human serum samples before and after *SHLI* injection. Clear separation was observed between the two groups, which indicated that the drug-induced constituents were contributed to the clustering.

### 3.3. Identification and Analysis of Prototype Components and Metabolites

**Figure 4:**
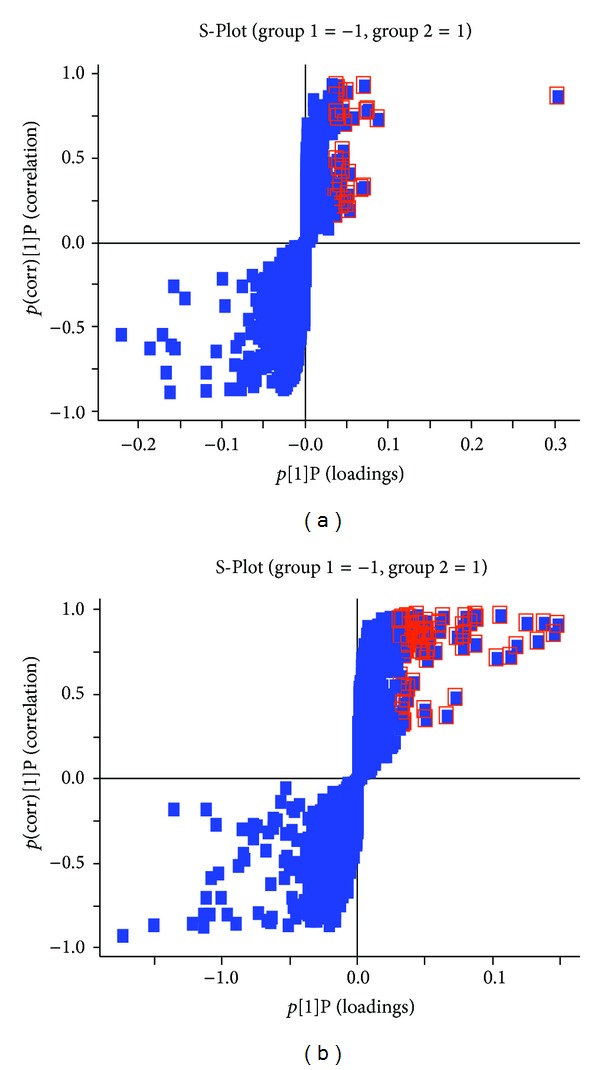
S-plots of human serum samples between C and *SHLI* groups in (a) positive ion mode and (b) negative ion mode. The ions marked with box were at the higher level in *SHLI* group.

In order to discover the multiple prototype components and metabolites of *SHLI* in human serum, S-plot, a tool for visualization and interpretation of multivariate classification models, was used. In the S-plot, each point represented an ion detected by UPLC-Q-TOF/MS. Variables that were the farthest from the origin in the S-plot were representative of the most significant changes between the two groups. Based on this, even subtle differences in the two groups could be easily extracted.  Figure 4 showed the ions in S-plot that were most responsible for distinguishing the C and *SHLI* groups and had a higher level in *SHLI* group.

**Figure 5:**
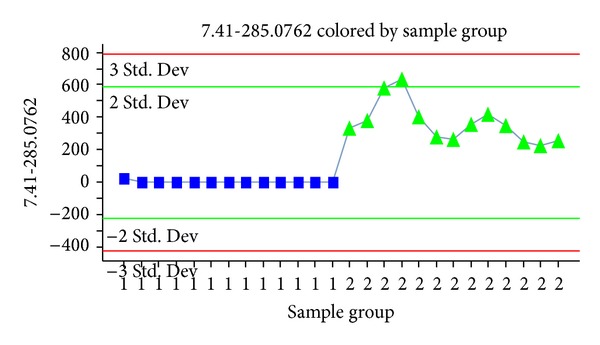
The trend plot of 7.41-285.0762 in positive mode between C group (■) and* SHLI* group (▲).

The S-plot responsible for the variances in the data was a combination of metabolites derived from the *SHLI* administration and endogenous molecules which were ubiquitous to serum and were interfered by *SHLI*. From a drug metabolite identification perspective, it was important that the disturbance endogenous molecules could be eliminated, and the prototype components and metabolites could be easily screened between *SHLI*-treated group and the control group. This comparison was achieved by using the trend plot. From the trend plots, the variables that only existed in the dosed serums were marked as the prototype components or the metabolites of *SHLI*.  Figure 5 showed the visualized trend plot of 7.41-285.0762 in positive mode between C group and *SHLI *group. The ion only appeared in the *SHLI* group. Therefore, 7.41-285.0762 might be a prototype component or a metabolite of *SHLI*.

Based on the metabolomic strategy, 35 exogenous components in human serum were found, among them, 23 prototype components of *SHLI* and 12 metabolites were identified and their information was shown in Table 2.

### 3.4. Characterization Analysis of Human Serum Prototype Components and Metabolites of *SHLI*

**Figure 6:**
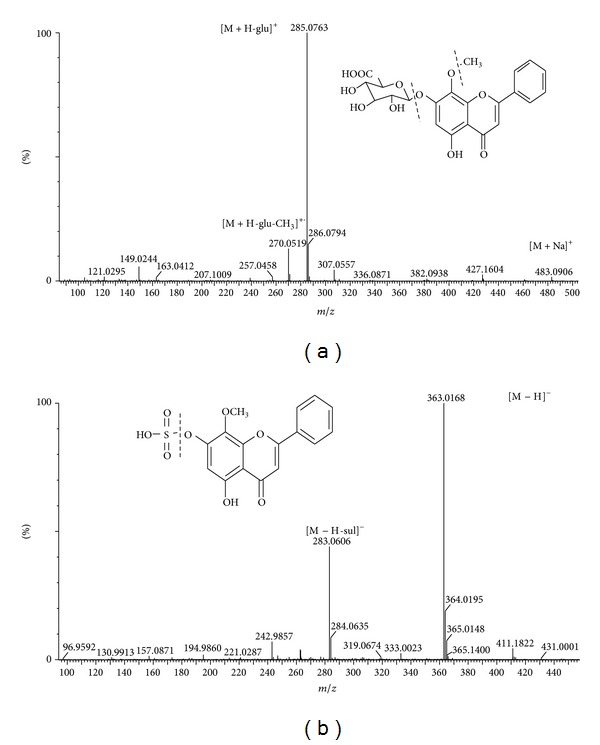
MS/MS spectra and structures of (a) prototype compound wogonoside in positive ion mode and (b) flavonoids metabolite wogonin 7-sulfate in negative ion mode identified in human serum after *SHLI* administration. In the tag, glu was the abbreviation of glucuronide-H_2_O and sul was the abbreviation of sulfate-H_2_O.

In our study, the prototype components and metabolites of *SHLI* were identified by comparing the accurate mass and MS^E^ fragment information obtained from the MS^E^ technique.  Figure 6 showed typical MS/MS spectra of the prototype component 6.23-461.1079 and the flavonoid metabolite 6.46-363.0174. In positive ion mode, the ion at *m/z* 483.0906 was [M + Na]^+^ion. The dominant fragment ion of *m/z *285.0763 was produced by loss of *m/z* 176 (glucuronide-H_2_O) fragment from [M + H]^+^ in positive ion mode. The characteristic and abundant fragment ion [M + H-CH_3_]^+•^ was generated by loss of CH_3_^•^ for the flavones with a methoxyl group on the side chains of an aromatic ring. Its molecular formula was speculated to be C_22_H_21_O_11_ based on the analysis of its elemental composition. Then the ion at *m/z* 483.0906 was inferred as wogonoside. The ion at *m/z* 363.0168 was [M − H] ^−^ ion. The major fragment ion of *m/z* 283.0606 was generated by loss of *m/z* 80 (sulfate-H_2_O) fragment from [M − H]^−^ in negative ion mode. The molecular formula was speculated to be C_16_H_12_O_8_S, and the fragmentation information and the molecular formula were consistent with wogonin 7-sulfate. Other metabolites were determined by the same method described above and some of them were also supported by the databases such as HMDB (http://www.hmdb.ca/) and METLIN (http://masspec.scripps.edu/). As a result, 23 prototype components and 12 metabolites of *SHLI* were identified.

### 3.5. Correlative Analysis of the Prototype Components and Metabolites of *SHLI*

The prototype herb components could be further metabolized by various drug metabolizing enzymes. Drug metabolism is classified into phase I and phase II reactions. Phase I reactions are mediated primarily by the cytochrome P450 family of microsomal enzymes [25]. Compounds are factionalized by oxidation, hydrolysis, or reduction, leading to the introduction of, for example, hydroxyl, amino, carboxyl, or thiol groups into the molecule. Most compounds undergo phase I oxidation prior to phase II conjugation, but molecules with sites amenable to conjugation may undergo phase II reactions directly. The most relevant phase II drug conjugation reactions are methylation, sulfation, glucuronidation, and glutathione conjugation. There were three types of components found in human serum after *SHLI* administration: (i) compounds found in their native form; (ii) phase I metabolites formed by chemical modifications, such as hydroxylation (M + OH) and hydration (M + H_2_O), and (iii) phase II metabolites formed by conjugation, such as methylation (M + CH_3_), glucuronidation (M + C_6_H_8_O_6_), sulfation (M + HSO_3_), and other conjugation reactions. In human serum, a large number of phase II metabolites were found. Among them, 8 flavonoids metabolites, 2 phenylephrine glycosides metabolites, 1 iridoid metabolite, and 1 quinic acid metabolite were found.

Some researchers have reported the metabolites of *SHL* formula in rat plasma [8]. We compared the metabolites differences in human and rats after *SHLI* administration and found great differences on the types and quantities of the metabolites after* SHLI* or* SHL* formula administrated between human and rats. The metabolites of *SHLI* found in rats and human were listed in Supplementary Material (see Table S1 in Supplementary Material available online at http://dx.doi.org/10.1155/2014/241505). Large number of phase I metabolites were detected in rats such as dihydrosecologanic acid and 3,4-dihydroxyphenylethanol, while little was found in the human serum. Besides, sulfated metabolites which were common in human serum were less detected in the rat plasma. Such a discrepancy might be attributed to different species (human or rats), prescription (*SHLI* or* SHL* formula), or blood collection time (1 h or shorter time). Further studies of the biological properties of these metabolites would be helpful to understand the pharmacological mechanism of* SHLI*.

## 4. Conclusion

In this paper, we developed an unbiased approach for screening the prototype components and metabolites of *SHLI *in human serum based on metabolomic technique. Employing UPLC-Q-TOF-MS combined with multivariate statistical analysis, 23 prototype components and 12 metabolites of *SHLI* were rapidly and sensitively identified, which suggested that the metabolomic approach was an effective tool to discover, screen, and analyze the multiple prototype components and metabolites from complicated traditional Chinese preparations in vivo. *SHLI* in human body mainly caused phase II metabolite reactions such as sulfation, methylation, glucuronidation, and other complex conjugation reactions. This identification and structural elucidation of the chemical compounds provided essential data for further pharmacological and pharmacokinetics study of *SHLI*. The human serum metabolomic approach avoids the laborious process of predicting possible metabolites and provides information on unexpected reactive metabolites and a type of validated rapid and higher throughput methodology for the identification of constituents of traditional Chinese medicine.

## Supplementary Material

amComparison of metabolites of *SHLI* and *SHL* formula found in human serum and rat plasma based on our research and previous reports.

## Figures and Tables

**Table 1:** UPLC-Q-TOF/MS identification of the constituents in *SHLI*.

NO.	*t*_*R*_ (min)	Positive ion MS	Negative ion MS	Formula	Identification	Positive ion MS/MS	Negative ion MS/MS	Class
1	0.88	193.0722	191.0557	C_7_H_12_O_6_	Quinic acid	112.0521	127.0400 85.0288	Quinic acid
2	3.77	355.1033	353.0873	C_16_H_18_O_9_	Chlorogenic acid	163.0395 145.0279 135.0454	191.0549 179.0341 135.0449	Quinic acid
3	3.87	—	375.1287	C_16_H_24_O_10_	Isomer of loganic acid	—	213.0765 169.0867 151.0759	Iridoid
4	3.90	623.2080	—	C_29_H_34_O_15_	Isomer of suspensaside A	191.0568 149.0232	461.1674 443.1554 205.0319	Phenylethanoid glycoside
5	3.97	—	461.1659	C_20_H_30_O_12_	Forsythoside E	—	315.1076 205.0718 135.0448	Phenylethanoid glycoside
6	4.20	—	375.1287	C_16_H_24_O_10_	Loganic acid	—	213.0778 169.0853 151.0773	Iridoid
7	4.24	355.1023	353.0866	C_16_H_18_O_9_	3-O-Caffeoylquinic acid	163.0393 145.0286	191.0569 179.0365	Quinic acid
8	4.29	—	353.0873	C_16_H_18_O_9_	4-O-Caffeoylquinic acid	—	173.0450 135.0453	Quinic acid
9	4.35	—	639.1925	C_29_H_36_O_16_	Suspensaside	—	621.1841 469.1273	Phenylethanoid glycoside
10	4.43	375.1288	373.1129	C_16_H_22_O_10_	Secologanic acid	213.0749 195.0638	193.0494 149.0605	Iridoid
11	4.45	391.1255	389.1074	C_16_H_22_O_11_	Monotropein	211.0586 177.0546 151.0395	209.0455 165.0554 149.0605	Iridoid
12	4.58	—	639.1918	C_29_H_36_O_16_	Isomer of suspensaside	—	445.1318 205.0318 179.0346	Phenylethanoid glycoside
13	4.72	—	403.1239	C_17_H_24_O_11_	Isomer of secoxyloganin	—	241.1177	Iridoid
14	4.73	359.1348	—	C_16_H_22_O_9_	Sweroside	197.0812 151.0400	—	Iridoid
15	4.80	625.2124	623.1982	C_29_H_36_O_15_	Acteoside	471.1504 325.0927 163.0398	461.1671 443.1567 203.0428	Phenylethanoid glycoside
16	4.89	—	755.2399	C_34_H_44_O_19_	Forsythoside B	—	593.2103 447.1500 315.1137	Phenylethanoid glycoside
17	4.93	623.1986	621.1816	C_29_H_34_O_15_	Suspensaside A	191.0571 149.0234	487.1371 469.1180	Phenylethanoid glycoside
18	5.03	625.2133	623.1970	C_29_H_36_O_15_	Forsythoside A	471.1512 325.0919 163.0398	461.1671 443.1567 205.0321	Phenylethanoid glycoside
19	5.06	405.1387	403.1236	C_17_H_24_O_11_	Secoxyloganin	243.0880 211.0612	371.0979 223.0611	Iridoid
20	5.09	—	515.1174	C_25_H_24_O_12_	3,4-Dicaffeoylquinic acid	—	353.0906 191.0561 135.0446	Quinic acid
21	5.13	—	515.1174	C_25_H_24_O_12 _	3,5-Dicaffeoylquinic acid	—	353.0906 173.0355 135.0446	Quinic acid
22	5.21	—	519.1863	C_26_H_32_O_11_	Pinoresinol 4-O-glucoside	—	357.1336 151.0398 136.0164	Lignan
23	5.30	463.0876	461.0730	C_21_H_18_O_12_	Luteolin 7-galacturonide	287.0552 269.0462 241.0493	285.0399 211.0400 113.0238	Flavonoid
24	5.32	—	447.0927	C_21_H_20_O_11 _	5,6-Dihydroxy flavanone-7-O-glucuronide	—	285.0399 267.0309 239.0356	Flavonoid
25	5.35	—	621.1788	C_29_H_34_O_15_	Suspensaside A	—	487.1510 179.0351	Phenylethanoid glycoside
26	5.37	611.1599	609.1453	C_27_H_30_O_16_	Rutin	465.1016 303.1489	300.0253 271.0236 255.0290	Flavonoid
27	5.38	517.1344	515.1186	C_25_H_24_O_12_	4,5-Dicaffeoylquinic acid	499.1206 355.1702 337.0856	353.0866 191.0553 173.0451	Quinic acid
28	5.54	—	757.2550	C_34_H_46_O_19_	Centauroside	—	525.1569 493.1695 179.0511	Iridoid
29	5.78	—	533.2020	C_27_H_34_O_11_	Phillyrin	—	371.1484 356.1257 121.0295	Lignan
30	5.88	447.0925	445.0771	C_21_H_18_O_11_	Baicalin	271.0603	269.0455 241.0503	Flavonoid
31	6.01	477.1027	475.0876	C_22_H_19_O_12_	5,2′-Dihydroxy-6′-methoxyflavone-7-O-glucuronide	301.0713	443.0556 299.0546	Flavonoid
32	6.17	431.0978	429.0815	C_21_H_18_O_10_	Chrysin 7-glucuronide	255.0658	253.0505	Flavonoid
33	6.23	461.1079	459.0927	C_22_H_20_O_11_	Wogonoside	285.0767 270.0534	283.0611 268.0375 239.0345	Flavonoid
34	6.38	—	445.0779	C_21_H_18_O_11_	Norwogonin-7-O-glucuronide	—	269.0422	Flavonoid
35	6.81	271.0608	269.0446	C_15_H_10_O_5_	Baicalein	271.0623 253.0498 123.0083	251.0362 223.0379 195.0447	Flavonoid
36	7.26	285.0761	283.0602	C_16_H_12_O_5_	Wogonin	270.0489	268.0377 162.9845	Flavonoid
37	7.35	255.0654	—	C_15_H_10_O_4_	Chrysin	153.0173	—	Flavonoid
38	7.40	285.0762	283.0601	C_16_H_12_O_5_	Isomer of wogonin	270.0489	268.0409	Flavonoid

**Table 2:** The prototype components and metabolites in human serum after *SHLI* dosed in both positive and negative mode.

NO.	*t*_*R*_ (min)	Positive ion MS	Negative ion MS	Formula	Identification	Positive ion MS/MS	Negative ion MS/MS	Relegation
1	0.88	193.0722	191.0557	C_7_H_12_O_6_	Quinic acid	112.0521	127.0400 85.0288	Prototype component
2	3.77	355.1033	353.0873	C_16_H_18_O_9_	Chlorogenic acid	163.0395 145.0279 135.0454	191.0549 179.0341 135.0449	Prototype component
3	3.87	—	375.1287	C_16_H_24_O_10_	Isomer of loganic acid	—	213.0765 169.0867 151.0759	Prototype component
4	4.20	—	375.1287		Loganic acid	—	213.0778 169.0853 151.0773	Prototype component
5	4.24	—	353.0873	C_16_H_18_O_9_	3-O-Caffeoylquinic acid	—	191.0569 179.0365	Prototype component
6	4.29	—	353.0873	C_16_H_18_O_9_	4-O-Caffeoylquinic acid	—	173.0450 135.0453	Prototype component
7	4.37	478.1365	—	C_22_H_23_NO_11_	Isorhamnetin 7-glucosamine	316.0847 298.0745 280.0654	—	Metabolite of flavonoids
8	4.42	—	475.1816	C_21_H_32_O_12_	Kanokoside A	—	313.0276 193.0493 123.0452	Metabolite of iridoids
9	4.43	375.1288	373.1129	C_16_H_22_O_10_	Secologanic acid	213.0749 195.0638	193.0494 149.0605	Prototype component
10	4.45	—	389.1074	C_16_H_22_O_11_	Monotropein	—	209.0455	Prototype component
11	4.57	—	369.0815	C_16_H_18_O_10_	Ferulic acid 4-O-glucuronide	—	193.0490 178.0263	Metabolite of quinic acids
12	4.72	—	403.1239	C_17_H_24_O_11_	Isomer of secoxyloganin	—	241.1177	Prototype component
13	4.73	359.1348	—	C_16_H_22_O_9_	Sweroside	197.0812 151.0400	—	Prototype component
14	5.03	—	731.1866	C_31_H_40_O_18_S	Methylated and sulfated forsythiaside	—	651.2212 457.1421	Metabolite of phenylethanoid glycosides
15	5.06	405.1387	403.1236	C_17_H_24_O_11_	Secoxyloganin	243.0880 211.0612	371.0979 223.0611	Prototype component
16	5.09	—	515.1174	C_25_H_24_O_12_	3,4-Dicaffeoylquinic acid	—	353.0906 191.0561 135.0446	Prototype component
17	5.16	623.1266	621.1092	C_27_H_26_O_17_	Genistein 4′,7-O-diglucuronide	447.0916 271.0607	445.0765 357.1336 269.0444	Metabolite of flavonoids
18	5.20	—	827.2600	C_37_H_48_O_21_	2-(3,4-Dihydroxyphenyl)ethyl6-deoxy-mannopyranosyl-glucopyranosyl-2-O-acetyl-4-O-[3-(3,4-dihydroxyphenyl)-2-propenoyl]-glucopyranoside	—	520.1801 429.1375 437.0904	Metabolite of phenylethanoid glycosides
19	5.21	—	519.1863	C_26_H_32_O_11_	Pinoresinol 4-O-glucoside	—	357.1336 151.0398 136.0164	Prototype component
20	5.47	623.1250	621.1088	C_27_H_26_O_17_	Baicalein 6,7-diglucuronide	447.0922 271.0605	445.0774 269.0452	Metabolite of flavonoids
21	5.54	609.1461	607.1299	C_27_H_28_O_16_	Luteolin 7-glucuronide-4′-rhamnoside	447.0919 271.0610	431.0965	Metabolite of flavonoids
22	5.54	—	757.2550	C_34_H_46_O_19_	Centauroside	—	525.1569 493.1695 179.0511	Prototype components
23	5.69	—	287.0234	C_11_H_12_O_7_S	5′-(3′,4′-Dihydroxyphenyl)-gamma- valerolactone sulfate	—	207.0651 179.0334 135.0437	Metabolite of flavonoids
24	5.78	—	533.2020	C_27_H_34_O_11_	Phillyrin	—	371.1484 356.1257 121.0295	Prototype component
25	5.88	447.0925	445.0771	C_21_H_18_O_11_	Baicalin	271.0603	269.0455 241.0503	Prototype component
26	6.17	431.0969	429.0815	C_21_H_18_O_10_	Chrysin 7-glucuronide	255.0645	253.0505	Prototype component
27	6.23	461.1079	459.0927	C_22_H_20_O_11_	Wogonoside	285.0760	283.0611 268.0375 239.0345	Prototype component
28	6.38	—	445.0779	C_21_H_18_O_11_	Norwogonin-7-O-glucuronide	—	269.0449 131.0625	Prototype component
29	6.41	—	349.0014	C_15_H_10_O_8_S	Baicalein 7-sulfate	—	269.0449	Metabolite of flavonoids
30	6.43	—	363.0174	C_16_H_12_O_8_S	Wogonin 7-sulfate	—	283.0606	Metabolite of flavonoids
31	6.46	—	283.0607	C_16_H_12_O_5_	7,5-Dihydroxy-6-methoxyflavone	—	268.0371	Metabolite of flavonoids
32	6.81	271.0608	269.0446	C_15_H_10_O_5_	Baicalein	271.0623 253.0498 123.0083	251.0362 223.0379 195.0447	Prototype component
33	7.26	285.0761	283.0602	C_16_H_12_O_5_	Wogonin	270.0489	268.0377 162.9845	Prototype component
34	7.35	255.0654	—	C_15_H_10_O_4_	Chrysin	153.0173	—	Prototype component
35	7.41	285.0761	283.0601	C_16_H_12_O_5_	Wogonin	270.0502	268.0409	Prototype component
