# Supplementary material for: A Metabolomic Strategy to Screen the Prototype Components and Metabolites of Shuang-Huang-Lian Injection in Human Serum by Ultra Performance Liquid Chromatography Coupled with Quadrupole Time-of-Flight Mass Spectrometry
Source: J Anal Methods Chem. 2014 Feb 26;2014:241505. doi: 10.1155/2014/241505 (PMC3955581; doi:10.1155/2014/241505)
Supplement: Supplementary file 1 — amComparison of metabolites of SHLI and SHL formula found in human serum and rat plasma based on our research and previous reports. [file 241505.f1.doc]

Table S1. The metabolites of *SHLI* or *SHL* formula found in human serum and rat plasma based on previous reports.

| Metabolic type | Metabolites | Human | Rat | Reactions |
| --- | --- | --- | --- | --- |
| Phase I  metabolites | Kanokoside A | + | - | Hydroxylation |
| Phase I  metabolites | Hydroxylpinoresinol | - | + | Hydrolysis and Hydrogenation |
| Phase I  metabolites | 3,4-Dihydroxyphenylethanol | - | + | Hydrogenation and hydrolysis |
| Phase I  metabolites | Demethyldihydrophillygenin  glucuronide | - | + | Demethylation |
| Phase I  metabolites | Dihydrosecologanic acid | - | + | Hydrogenation |
| Phase I  metabolites | 3,4-Dihydroxyphenylethanol | - | + | Hydroxylation |
| Phase I  metabolites | p-Hydroxyphenyl-propionic acid | - | + | Hydrolysis |
| Phase I  metabolites | Hydroxylpinoresinol | - | + | Hydroxylation |
| Phase II metabolites | 3,4-Dihydroxyphenylglycol  sulfate | - | + | Hydrogenation and  sulfation |
| Phase II metabolites | 2-(3,4-Dihydroxyphenyl)  ethanol sulfate | - | + | Sulfation |
| Phase II  metabolites | 7,5-Dihydroxy-6-methoxyflavone | + | - | Methylation |
| Phase II  metabolites | Wogonin 7-sulfate | + | - | Sulfation |
| Phase II  metabolites | Baicalein 7-sulfate | + | - | Sulfation |
| Phase II  metabolites | 5'-(3',4'-Dihydroxyphenyl)-gamma-valerolactone sulfate | + | - | Sulfation |
| Phase II  metabolites | Luteolin 7-glucuronide-4'-rhamnoside | + | - | Polysaccharide  conjugation |
| Phase II  metabolites | Baicalein 6,7-diglucuronide | + | - | Glucuronidation |
| Phase II  metabolites | 2-(3,4-Dihydroxyphenyl)ethyl6-deoxy-  mannopyranosyl-glucopyranosyl-2-O-acetyl-4-O-[3-(3,4-dihydroxyphenyl)-2-propenoyl]-glucopyranoside | + | - | Polysaccharide  conjugation |
| Phase II  metabolites | Genistein 4',7-O-diglucuronide | + | - | Glucuronidation |
| Phase II  metabolites | Methylated and sulfated forsythiaside | + | - | Methylation and sulfation |
| Phase II  metabolites | Ferulic acid 4-O-glucuronide | + | - | Glucuronidation |
| Phase II  metabolites | Isorhamnetin 7-glucosamine | + | - | Methylation and glucosamination |
| Phase II  metabolites | Dihydroxy-trimethoxyflavanone | - | + | Methylation |
| Phase II  metabolites | Dihydroxy-dimethoxy flavone  glucuronide | - | + | Methylation |

+ Represents found in human serum or rat plasma.

- Represents unfound in human serum or rat plasma.
